# Camel Brucellosis: Seroprevalence, Associated Risk Factor, and Public Health Perceptions in Arero District of Borena Zone, Southern Ethiopia

**DOI:** 10.1155/vmi/3722054

**Published:** 2025-01-10

**Authors:** Wario Waji Edema, Gemechu Chala Hunderra, Sultan Abda Neja

**Affiliations:** Faculty of Veterinary Medicine, Hawassa University, P.O. Box 05, Hawassa, Ethiopia

**Keywords:** Arero, brucella, camel, public perception, risk factor, seroprevalence

## Abstract

A cross-sectional study was conducted to determine the seroprevalence and potential risk factors of camel brucellosis and to assess public health awareness of the disease in the selected kebele of Arero District, Borena Zone, Southern Ethiopia. A total of 313 blood samples were collected from selected camels using a systematic random sampling technique. The serum samples underwent initial screening for brucellosis using the rose Bengal plate test (RBPT), with further confirmation through the indirect enzyme-linked immunosorbent Assay (i-ELISA). The overall seroprevalence of camel brucellosis was 7.66% using RBPT and 2.24% using i-ELISA. Univariable and multivariable logistic regression analyses revealed significant associations; female camels (OR = 30.16, *p*=0.05) and mixing camels with other animal species (OR = 0.019, *p*=0.001 by i-ELISA) were significantly linked to camel seropositivity for Brucella infection. Assessment of public health perception and awareness from 227 owners indicated limited awareness; most respondents lacked knowledge about brucellosis (94.27%), 82.82% were unaware of raw meat risks, 94.27% did not know about brucellosis being zoonotic, and 18.94% consumed raw milk with blood. The majority (94.71%) practiced bare-handed calving, with 93.39% handling abortion materials, rarely isolating infected camels. The study found a moderate prevalence of brucellosis among camels in the study areas. Lack of implemented control strategies, combined with local pastoral practices, could potentially contribute to disease spread. Therefore, there should be continuous efforts of the disease control strategy at the animal level, community awareness creation, separation of infected camels from health heard, and avoiding mixing a camel with other animal species.

## 1. Introduction

Camels are versatile, vital domestic animals that are best adapted to dry environmental conditions prevailing in extreme semi-arid and arid areas worldwide. They are endowed with extremely extraordinary features that enable them to survive and perform under extremely harsh conditions [1]. According to the Food and Agriculture Organization (FAO) Corporate Statistical Database (FAOSTAT), Somalia has the highest camel population globally, followed by Sudan, Kenya, and Ethiopia [2]. According to the recent reports of central statistics agency of Ethiopia, the current camel population in Ethiopia is estimated to be more than 2.4 million [3, 4].

Camels are integral to Ethiopia's pastoral regions, particularly in adapting to environmental challenges such as droughts and land degradation [5]. They thrive in arid areas, supporting livelihoods by providing milk, food, income, and transportation, even on less fertile lands [6]. Their extended lactation and consistent milk production during dry spells enhance their economic value [7, 8].

Despite its advantages, camel farming faces significant challenges, particularly infectious diseases such as brucellosis, which impacts both animal and human health [9, 10]. Brucellosis, caused mainly by *Brucella abortus* and *Brucella melitensis*, is a widespread zoonosis prevalent in Africa, Asia, the Mediterranean, and the Middle East [11–17]. In Ethiopia, it causes economic losses due to reduced milk production, infertility, and reproductive issues such as abortion and delayed calving [18–23]. A significant economic loss due to camel brucellosis was attributed to abortion or perinatal mortality of calves (341,325 ETB) in the Elwayye District, southern Ethiopia [20]. In a separate study conducted in the Mehoni District, southeastern Tigray, Ethiopia, the estimated economic loss caused by camel brucellosis was calculated at 429,351.48 ETB (21,467.56 USD) per infected camel aged over 4 years [21]. These pilot assessments highlight a substantial economic burden on the pastoral community and the national livestock industry as a whole. The disease often spreads when camels interact with infected ruminants, with bacteria entering through inhalation, ingestion, or mucous membranes, leading to inflammation and reproductive failure [15, 17, 24].

The impact of brucellosis is concerning in pastoral regions due to limited awareness of its zoonotic importance [25]. Recognized by FAO, World Health Organization (WHO), and World Organization for Animal Health (WOAH), camel brucellosis stands as a highly contagious and economically significant zoonotic disease globally [26]. Infected camels and livestock pose risks to human populations through contact with birthing materials, consumption of unpasteurized dairy, among other routes. Pastoralists in these areas face heightened infection risks due to the absence of disease control strategies [18, 27].

Camel husbandry thrives in Ethiopia's eastern and southern regions, such as Afar, Somali, and Borena, sustaining pastoral communities' livelihoods. Brucellosis, a significant economic and public health challenge in these areas, has been reported with varying prevalence across different agroecologies. Previous studies reported 2.4% prevalence in the Somali region [28] and 2.09% in other parts of Ethiopia [29]. Higher rates were observed in Afar (5.4%) and southeastern Tigray (3.67%) [30, 31], while lower rates were noted in the Borena Zone (0.9%) [32], Tigray (1.5%) [33], and Hararghe regions (1.53%) [34]. At the herd level, this study found seroprevalence rates of 0.0%, 33.3%, and 23.8% for small, medium, and large herds, respectively, aligning with the 16% herd-level prevalence reported by Bekele in the Borena lowlands [35] but exceeding 6.9% reported by Jara et al. in the same zone [36]. Variations in prevalence may stem from ecological differences, herd management practices, diagnostic methods, and increased cross-species contact, particularly during calving or abortion periods, which heighten the risk of *Brucella* spread [37]. Effectively controlling camel brucellosis requires diagnostic and surveillance systems and evaluating cost-effective control measures [38, 39].

Despite a substantial camel population in Ethiopia's pastoral areas, information on camel brucellosis prevalence and management practices is still limited [28, 39]. Disease control efforts are lacking in many regions, reflecting a research gap with scarce published reports on camel brucellosis prevalence. On top of economic loss, close human-animal contact and raw animal product consumption pose significant zoonotic risks, particularly in pastoral regions, calling for thorough epidemiological investigations to identify key risk factors influencing disease occurrence. A study on the seroprevalence and risk factors for human brucellosis in pastoralists in Afar, Ethiopia, revealed a 16.5% seroprevalence of brucellosis among 91 human serum samples analyzed. Notably, 86.7% of the positive cases were female, and the highest proportion of seropositive individuals was observed in those under 15 years old [40]. Another study on zoonotic risks of camel brucellosis in Afar region showed 7.6% seroprevalence of brucellosis in camel and 10% of camel owners for which occupation and nonprotective handling of dystocia cases significantly association with *Brucella* seropositivity [41]. In both studies, most cases were linked to close contact with animals, consumption of raw milk, and handling parturient materials, underscoring the zoonotic risk of brucellosis in camel-producing areas of Ethiopia. Therefore, the present study investigates the seroprevalence, associated risk factors, and public health perception of camel brucellosis in Arero District of Borena Zone, Southern Ethiopia.

## 2. Materials and Methods

### 2.1. Study Area

**Figure 1:**
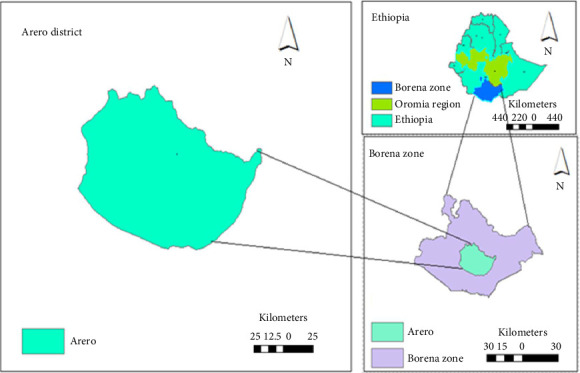
Location map of the study area [42].

Arero District, situated approximately 100 km from Yabello town along the Addis Ababa–Moyale main road, is part of the Borena Zone in the Oromia region (Figure 1). It shares borders with Dubuluk, Yabello, Gomole, the Guji zone, Somali region, Wachile District, and Das District, separated by the Dawa Ganale River. The district spans altitudes from 710 to 1789 m above sea level. Its population consists of approximately 95,404 individuals, primarily rural (89.5%), with an urban population of 10.5%. Covering an area of about 10,841.88 square kilometers, Arero hosts a livestock population including 44,193 camels, 193,693 cattle, 110,585 goats, and 49,691 sheep. The study included 313 camels during this period, as reported by the Arero District Livestock and Resources Development Bureau (ADLRDB) in 2020 [42].

### 2.2. Study Population

The study population was indigenous camel breeds (Borena breed) of both sexes with 6 months or above age that were kept under traditional (extensive) management systems in the eight peasant associations of the district.

### 2.3. Sample Size Determination

The required sample size (*n*) was determined using the 3% previous prevalence in the relative area of the study area [36] and 95% confidence interval with 5% precision according to Thrusfield [43]. Therefore, the calculated sample size was 45; however, the calculated sample size was increased to 313 to improve the study precision and decrease the possible bias. Hence, a total of 313 camels were considered for this study from selected kebeles of Arero District.

### 2.4. Sampling Technique

In the present study, multistage sampling was used to address the logistical challenges and lack of comprehensive sampling frames associated with the nomadic nature of pastoral production systems. According to Dohoo et al. [44], this method enabled the selection of samples through hierarchical stages, starting from regions to communities and finally individual herds or animals, ensuring feasibility, cost-effectiveness, and statistical rigor. The district was purposively selected based on accessibility, distribution of the camel population, occurrences of abortion outbreaks, and the absence of specific research on camel brucellosis in the study area. The purposive selection of the district based on accessibility, camel distribution, abortion outbreaks, and the absence of prior research enhances the external validity of the study by ensuring that the study area represents key characteristics of camel-rearing pastoral systems with a high likelihood of brucellosis presence. These factors improve the relevance of the findings to similar pastoral settings and populations, allowing for broader generalizability while addressing gaps in the existing research.

After districts were selected, kebeles, villages, and herds were chosen using simple random sampling techniques, while individual animals were selected through systematic random sampling. Kebeles and villages were considered primary units, herds as secondary units, and individual animals as tertiary units. As per the local animal office, the number of villages in each kebele varied from 8 to 10, with 18–30 households within each village. The herds in the selected villages were listed with the assistance of local animal health workers. A total of 76 herds existed in all eight selected kebeles, of which 42 were randomly chosen from Guto (7 herds), H/dimtu (4), K/gumata (6), Renji (6), Oroto (4), Silala (5), Fuldowa (5), and Bobele (5). Subsequently, four to seven animals were selected using systematic random sampling from each of the chosen herds, encompassing both sexes and camels above 6 months of age. Information regarding individual animals, including sex, age, body condition, abortion history, physiological status, retention of fetal membrane, interaction with other species, and parity, was recorded. To assess the pastoralists' disease awareness, 227 pastoral attendants from the aforementioned herds who agreed to be interviewed participated in the study.

### 2.5. Sample Collection and Laboratory Analysis

Ten milliliters of blood were collected from each animal's jugular vein using sterile needles and plain vacutainer tubes. Samples stood overnight at room temperature, and sera were decanted into cryovials, transported to the Yabello Regional Veterinary Laboratory in ice packs, and stored at −20°C for antibody screening.

#### 2.5.1. Serological Laboratory Techniques

Two serological tests were sequentially applied for increased accuracy: RBPT served as a screening tool due to cost-effectiveness and high sensitivity, while i-ELISA provided high specificity and distinguished false-positive cross-reactions from *Brucella* infections [45]. RBPT was performed on collected sera using commercially prepared *Brucella* antigens following WOAH procedures [46]. Agglutination reactions were observed and classified as positive or negative. RBPT-positive samples underwent confirmation using i-ELISA, a commercially available assay employing *B. abortus* antigens. Results were expressed as an OD ratio for confirmation.

### 2.6. Questionnaire Survey

A semistructured questionnaire was developed based on previous studies that assessed the seroprevalence, associated risk factors, and zoonotic transmission risks of brucellosis from camels to humans [36, 41, 47]. The collected information covers animal-related risk factors such as animal age, history of abortion, contact with other ruminants, experience in camel rearing, and public risk factors including the consumption of raw milk, handling of aborted fetuses, exposure to vaginal discharges from infected camels, and knowledge of brucellosis and its zoonotic significance. Due to technical limitation of professionals to collect human samples, the present study did not investigate disease status in humans.

### 2.7. Data Management and Analysis

Data from both surveys and laboratory tests were recorded in Microsoft Excel and analyzed using STATA 9.0. Seroprevalence was determined, and associations between *Brucella* seropositivity and various risk factors were explored through multivariable logistic regression, considering variables with a *p* value ≤ 0.05 as significantly associated with brucellosis.

## 3. Results

### 3.1. Seroprevalence of Camel Brucellosis

Out of the 313 serum samples examined, 24 (7.7%) of them were found to be positive for brucellosis by the RBP screening test. However, of the 24 RBPT positive sera, only 7 (2.2%) were confirmed with the i-ELISA. Therefore, the overall individual-level seroprevalence of camel brucellosis in the study was found to be 2.2% (Table 1).

In terms of locality, camel brucellosis seropositivity was notably the highest in H/Dimtu (10.0%), followed by Fuldowa (5.7%) and Renji (3.3%), while none of the other studied kebeles (Silala, Guto, K/Gumata, Bobela, and Oroto) tested positive based on the i-ELISA results. This outcome showed statistical significance prevalence among the localities (*p*=0.027). A significantly higher seroprevalence of camel brucellosis in male animals compared to female animals was recorded (*p*=0.014), while comparable seroprevalence was observed among animals with medium and good body condition (Table 1). Among the 42 examined camel herds, 7 tested positive using the i-ELISA test. Thus, the overall herd-level seroprevalence of camel brucellosis in the study area was 16.7% (Table 2).

### 3.2. Analysis of Risk Factors for Camel Brucellosis Seropositivity

Univariable analysis was undertaken for the 11 explanatory variables to see their association with camel brucellosis seropositivity (Table 3). Accordingly, the results of the univariable logistic regression analysis identified six risk factors to be significantly (*p* < 0.05) associated with seropositivity of camel brucellosis. However, there was no significant (*p* > 0.05) association observed between camel brucellosis seropositivity and the rest five explanatory variables (Table 3).

In the multivariable logistic regression model, no associations were found between camel brucellosis seropositivity and abortion history and parity (Table 4).

The multivariable regression analysis revealed that the odds of camel seropositivity for brucellosis was higher in female camels (*p*=0.022; OR: 8.11; 95% CI: 1.35–48.72), in camels reared with cattle (*p*=0.033; OR: 6.45; 95% CI: 1.17–35.71) and small ruminants (*p*=0.002; OR: 14.48; 95% CI: 2.56–81.79), and in camels with a history of retained fetal membrane (*p*=0.016; OR: 0.61; 95% CI: 0.09–3.94) than their counter parts (Table 4).

### 3.3. Camel Owners' Awareness About Camel Brucellosis and Zoonosis

Questionnaire data were collected from 227 camel owners to assess their awareness regarding camel brucellosis and other zoonotic diseases. According to the data, the majority (94.27%) of camel owners had no information about brucellosis at both human and animal levels. Approximately 91.2% of the respondents reported habitual consumption of raw milk, with 18.9% consuming milk with blood. Moreover, roughly 82.8% of the respondents admitted to consuming raw or undercooked meat (Table 5). Remarkably, the majority (93.39%) of camel owners handled abortion and aborted material without adopting productive measures, while only 6.61% mentioned using protective materials such as plastic when dealing with abortion and aborted material. Furthermore, about 5.3% of camel owners had a practice of separating aborted camels from the herd, whereas the majority (94.71%) did not segregate aborted or diseased camels from the healthy herd. In addition, only a small fraction (5.3%) of the respondents were known to use protective materials when assisting animals during parturition (Table 5).

## 4. Discussion

Despite existing data on *Brucella* infections in humans and animals exist for Ethiopia and the Borena Zone [23, 48, 49], camel brucellosis status in our study area remains poorly understood. Our study detected camel brucellosis serologically in eight selected kebeles within the Arero District of the Borena pastoral area, Ethiopia. The 2.2% prevalence of camel brucellosis confirmed by iELISA in our study closely aligns with 2.4% and 2.09% rates reported in the Somali region [28] and other areas of Ethiopia [29] while slightly lower than the 3.7% recently reported in Elwayye District of Borena Zone [20]. However, our result was lower than rates reported in Afar and southeastern Tigray regions (5.4% and 3.67%, respectively) [30, 31], although still higher than the former report in the Borena Zone [32], Tigray [33], and Hararghe regions [34]. Similarities in prevalence might be due to shared pastoralist practices across the country while the differences could stem from various factors such as geographical, climatic variations, study design, and population differences [32, 33, 50]. It is worth mentioning that management systems, diagnostic tests used, and sampling strategies, as these could provide deeper insights into the discrepancies in prevalence rates of camel brucellosis.

As it is importantly accepted that brucellosis is considered as a disease of herd importance, coincidently, the present study revealed considerably higher prevalence of camel brucellosis at the herd level with a seroprevalence of 0.0%, 33.3%, and 23.8%, in small, medium, and large herd sizes, respectively. The 16.7% camel brucellosis seroprevalence observed in the current study was in accord with the 16% herd level seroprevalence previously reported from Borena lowlands by Bekele [35] and higher than the 6.9% previous study report from Borena Zone by Jara et al. [36]. The observed difference in the herd-level seroprevalence of camel brucellosis might be related to the actual individual animal–level prevalence of the disease and/or the variation in the actual herd size studied in the different studies [33, 34, 50]. The higher herd-level prevalence found in this study might be due to increased interaction between camel herds and other animals, particularly ruminants, in the study areas. Herding camels alongside cattle, sheep, and goats could heighten the chances of cross-species contact, potentially leading to *Brucella* spread, especially during calving or abortion periods when brucellosis contamination is more likely [37]. Increased interaction between camel herds and other ruminants, such as cattle, sheep, and goats, elevates the risk of *Brucella* transmission. A review on camel brucellosis highlights herd size and cohabitation with other ruminants as key risk factors [18]. Additional factors include increased species composition at the household level and the wet season [51]. Camels are infected through spillover from small ruminants and cattle [52]. A study on brucellosis in camels, small ruminants, and Somali pastoralists found that small ruminants from large herds were 5.01 times more likely to acquire *Brucella* spp. infection compared to those in smaller herds [52]. These findings suggest that an increased interaction between camel herds and other ruminants contributes to higher herd-level camel brucellosis prevalence.

Variations in seroprevalence across studies could also stem from test differences in sensitivity and specificity, agroecological variations, sample sizes, production systems, and study periods [53]. Understanding risk factors is a key for brucellosis control. Factors such as animal age, sex, breed, farm management, and farmer awareness impact brucellosis prevalence [54]. In our study, larger herd sizes showed more *Brucella* antibodies, likely due to increased transmission risk with higher stocking density postabortion or calving [55, 56]. However, antibody distribution among kebeles, animal age, body condition, physiological status, and parity remained consistent. This might relate to similar sample sizes across kebeles and could reflect the overall seroprevalence in the area and studied animals.

In the current study, animal's sex was significantly associated with brucellosis seropositivity in which male animals are more likely (OR: 8.11) to be seropositive for brucellosis than females (*p*=0.022; 95% CI: 1.35–48.72). This finding was in agreement with the previous report from Yabello District of the Borena Zone by Admasu and Kaynata [16, 57]. Conversely, Jara et al. [36] revealed that the likelihood of camel brucellosis seropositivity is higher in female animals than in males. The observed differences might be related to the difference in the management system, number of female and male animals in the herd, and serological tests employed among others [33, 34, 50]. Moreover, it was evidenced that female animals are generally more susceptible to brucellosis than males due to the fact that female animals are more under physiological stress and also could be attributed to the higher concentration of the sugar erythritol in females than in males [58]. Hence, the current result should be explained cautiously with potential biases due to the small number of male animals considered during the study period compared to their female counters.

The study also noted a diverse presence of camels, sheep, goats, and cattle in the area, potentially increasing disease transmission between these animals [35, 54]. Camels in herds with sheep and goats contact showed significantly higher brucellosis rates, more than 14 times the risk of those without such contact (*p*=0.002; OR: 14.48; 95% CI: 2.56–81.79). Similarly, contact with cattle also increased brucellosis risk for camels (*p*=0.033; OR: 6.45; 95% CI: 1.17–35.71). This might be due to shared pasture and watering points, enabling transmission from ruminants to camels, aligning with previous Ethiopian and international studies [23, 30, 48, 59, 60].

The increased risk of camel brucellosis transmission through contact with other animals such as cattle, sheep, and goats is well documented. Studies have showed that *Brucella* spp., particularly *B. melitensis* and *B. abortus*, can be transmitted between these species through direct contact or shared environments [52, 61]. Shared grazing lands and water sources are common in pastoral systems, facilitating the spread of the bacteria [62]. For instance, the same *B. melitensis* biovars were cultured from both camels and sheep sharing the same premises [63]. In addition, vectors such as ticks and flies can play a role in the transmission of brucellosis between different livestock species [52, 64]. These factors underscore the importance of integrated disease management strategies to control brucellosis across multiple species in pastoral communities.

The findings from the questionnaire survey highlight a critical lack of awareness among camel owners regarding the public health risks associated with brucellosis, which has serious implications for both animal and human health. The survey revealed that 82.82% of participants were unaware of the risks of consuming raw meat, and an alarming 94.27% lacked awareness of the zoonotic nature of brucellosis. These gaps in knowledge are particularly concerning in pastoral communities where traditional practices, such as consuming raw milk mixed with blood (reported by 18.94% of participants), are prevalent. The survey also uncovered that 94.71% of animal owners assisted with calving bare-handed, and 93.39% handled abortion materials without any protective measures. Such behaviors increase the risk of *Brucella* transmission both within camel herds and to humans, as infected birth products are known to be highly contagious [62, 65].

The lack of knowledge regarding the zoonotic nature of brucellosis is a significant public health concern. Brucellosis in humans, often referred to as undulant fever, can lead to debilitating chronic illnesses characterized by fever, joint pain, and fatigue, and it is frequently misdiagnosed due to its nonspecific symptoms [66]. This is particularly worrisome in pastoralist communities where raw milk and meat consumption is common, as these are primary transmission routes for *Brucella* spp. [64]. Studies from other regions, such as in Kenya and Sudan, with similar pastoralist lifestyles corroborate these findings, showing that human brucellosis cases are strongly associated with handling infected animals and consuming raw animal products [19, 62, 67].

The implications of these knowledge gaps are profound. They likely contribute to the ongoing transmission of brucellosis within camel herds and to humans, perpetuating a cycle of infection that is difficult to break without targeted interventions. Education campaigns tailored to pastoralist communities, emphasizing the zoonotic risks of brucellosis and the importance of protective measures, such as wearing gloves during calving and avoiding raw milk and meat, could significantly mitigate these risks. For example, community-based training programs in Tanzania have successfully improved awareness and reduced risky behaviors related to zoonotic diseases [68]. Veterinary outreach programs that include routine testing and isolation of infected animals, coupled with improved veterinary infrastructure, could further reduce transmission.

Comparisons with other regions revealed that knowledge gaps about brucellosis are widespread in pastoral settings, but the extent varies. In the Lega Hida area of Bale Pastoralist of Ethiopia, studies reported similar low awareness levels, with only 17% of livestock keepers knowing about zoonotic risk of camel brucellosis [22]. This underscores the need for region-specific strategies that address cultural practices and local epidemiological factors. Expanding public health initiatives and integrating brucellosis awareness into broader zoonotic disease education programs are essential steps toward improving both animal and human health outcomes in these communities.

## 5. Conclusion

The study identified a moderate seroprevalence of camel brucellosis in the Borena Zone, which is higher than previously reported findings in the region. While acknowledging the study's methodological limitations, it successfully identified key risk factors associated with the disease's prevalence. In addition, the findings highlighted significant gaps in community awareness regarding risky practices, such as consuming raw meat and milk, bare-handed handling of newborns and abortion materials, all of which contribute to the potential zoonotic transmission of brucellosis. These results underscore the importance of addressing camel brucellosis as a public health and livestock management concern in the region. To mitigate its impact, we recommend further comprehensive epidemiological studies, improved camel health management practices, community-based awareness campaigns on disease transmission risks, and the implementation of measures to isolate infected animals within herds.

## Figures and Tables

**Table 1:** Seroprevalence of camel brucellosis stratified by locality, age, and sex.

Variables	Category	No. of animals examined	No. of (%) animals positive		95% CI for RBPT	95% CI for i-ELISA
			RBPT	i-ELISA		
Kebele	Silala	39	7 (17.9)	0 (0.0)	11.5–28.8	0.0–10.4
	Guto	39	0 (0.0)	0 (0.0)	0.0–10.4	0.0–10.4
	H/Dimtu	30	3 (10.0)	3 (10.0)	3.4–21.3	3.4–21.3
	K/Gumata	40	0 (0.0)	0 (0.0)	0.0–10.9	0.0–10.9
	Renji	30	2 (6.7)	1 (3.3)	1.6–17.4	0.2–14.5
	Bobela	40	3 (7.5)	0 (0.0)	1.9–19.1	0.0–10.4
	Fuldowa	53	5 (9.4)	3 (5.7)	3.7–21.2	1.5–16.6
	Oroto	51	4 (7.8)	0 (0.0)	2.6–16.3	0.0–10.9

Sex	Female	274	19 (96.9)	4 (1.5)	94.0–99.2	0.7–3.8
	Male	39	5 (12.8)	3 (7.7)	4.0–26.4	1.2–23.1

Age	Young	73	7 (9.6)	3 (4.1)	3.4–19.2	0.7–13.3
	Adult	240	17 (7.1)	4 (1.7)	4.0–11.1	0.7–4.1

BCS	Poor	74	5 (6.8)	0 (0.0)	2.5–14.2	0.0–8.8
	Medium	169	13 (7.7)	4 (2.4)	4.8–12.7	1.0–6.3
	Good	70	6 (8.6)	3 (4.3)	3.8–16.3	1.2–12.6

Abbreviations: BCS, body condition score; CI, confidence interval; i-ELISA, indirect enzyme-linked immunosorbent assay; RBPT, rose Bengal plate test.

**Table 2:** Herd-level seroprevalence of camel brucellosis.

Variables	Category	No. of herds examined	No. of animals examined	No. (%) of herds positive	No. (%) of animals positive	
				i-ELISA	RBPT	i-ELISA
Herd	Small	12	29	0 (0.0)	1 (3.4)	0 (0.0)
	Medium	9	73	2 (33.3)	4 (5.5)	2 (2.7)
	Large	21	211	5 (23.8)	19 (9.0)	5 (2.4)

Abbreviations: i-ELISA, indirect enzyme-linked immunosorbent assay; RBPT, rose Bengal plate test.

**Table 3:** Univariable logistic regression analysis of potential risk factors for camel *Brucella* seropositivity by i-ELISA.

Potential risk factors	Category	No examined	No. (%) of animals positive	B	*p* value	OR (95% CI)
Sex	Female	274	4 (1.5)	−1.727	0.028	0.18 (0.04–0.83)
	Male	39	3 (7.7)			

Age	Young	73	3 (4.1)	0.928	0.232	0.40 (0.09–1.81)
	Adult	240	4 (1.7)			

Kebele	Silala	39	0 (0.0)		0.993	Ref.
	Guto	39	0 (0.0)	19.01	0.998	—
	H/Dimtu	30	3 (10.0)	19.01	0.998	—
	K/Gumata	40	0 (0.0)	19.01	0.998	—
	Renji	30	1 (3.3)	−1.170	0.324	0.31 (0.03–3.17)
	Bobela	40	0 (0.0)	19.01	0.998	—
	Fuldowa	53	3 (5.7)	−0.616	0.469	0.54 (0.10–2.86)
	Oroto	51	0 (0.0)	19.01	0.998	—

BCS	Poor	74	5 (6.8)	—	0.732	Ref.
	Medium	169	13 (7.7)	18.1	0.997	—
	Good	70	6 (8.6)	−0.614	0.430	0.54 (0.12–2.48)

Abortion history	Yes	143	2 (1.4)	0.895	0.076	2.45 (0.91–6.57)
	No	131	2 (1.5)			

Contact with cattle	Yes	31	3 (9.7)	2.008	0.011	7.45 (1.59–34.96)
	No	282	4 (1.4)			

Contact with sheep and goat	Yes	37	4 (10.8)	2.401	0.002	11.03 (2.37–51.45)
	No	276	3 (1.1)			

Herd size	Small	12	0 (0.0)	—	0.985	Ref.
	Medium	9	2 (33.3)	17.33	0.981	—
	Large	21	5 (23.8)	0.149	0.861	1.16 (0.22–6.12)

Retained fetal membrane	Yes	42	3 (7.1)	−2.877	0.014	0.06 (0.01–0.56)
	No	232	1 (0.4)			

Parity	< 3	133	1 (0.75)	−2.678	0.018	0.07 (0.01–0.63)
	≥ 3	241	2 (1.4)			

Physiological status	Dry off	139	1 (0.7)	—	0.137	Ref.
	Lactating	83	1 (1.2)	0.513	0.718	1.67 (0.10–27.07)
	Pregnant	52	2 (3.8)	1.701	0.169	5.48 (0.49–61.76)

*Note:* B, estimate.

Abbreviations: BCS, body condition score; CI, confidence interval; OR, odds ratio; Ref., reference; RFM, retained fetal membrane.

**Table 4:** Multivariable logistic regression analysis of significantly associated explanatory variables for camel *Brucella* seropositivity.

Explanatory variables	Category	No. of observations	No. (%) of positive with i-ELISA	B	*p* value	OR (95% CI)
Sex	Female	274	4 (1.5)	2.093	0.022	8.11 (1.35–48.72)
	Male	39	3 (7.7)			

Contact with sheep and goat	Yes	37	4 (10.8)	2.672	0.002	14.48 (2.56–81.79)
	No	276	3 (1.1)			

Contact with cattle	Yes	31	3 (9.7)	1.864	0.033	6.45 (1.17–35.71)
	No	282	4 (1.4)			

RFM	Yes	42	3 (7.1)	−0.498	0.016	0.61 (0.09–3.94)
	No	232	1 (0.4)			

*Note:* B, estimate.

Abbreviations: CI, confidence interval; i-ELISA, indirect enzyme-linked immunosorbent assay; OR, odds ratio; RFM, retained fetal membrane.

**Table 5:** Knowledge, understanding, and practice of camel owners regarding to camel brucellosis.

Assessments of awareness of owner	Category	Freq.	(%)
Consume raw meat	Yes	39	17.18
	No	188	82.82

Awareness	Yes	13	5.73
	No	214	94.27

Consume milk with blood	Yes	43	18.94
	No	184	81.06

Handle abortion material	Yes	212	93.39
	No	15	6.61

Separate aborted animals	Yes	12	5.29
	No	215	94.71

Assist during calving	Yes	215	94.71
	No	12	5.29

Consume raw milk	Yes	207	91.19
	No	20	8.81

## Data Availability

The data that support the findings of this study are available from the corresponding author upon reasonable request.

## Nomenclature

AMOS: *Brucella abortus*, *melitensis*, *ovis*, and *suis*
CDC: Center for Disease Control
CFT: Complement fixation test
CSA: Central Statistical Authority
i-ELISA: Indirect enzyme-linked immunosorbent assay
FAO: Food and Agriculture Organization
IgG: Immunoglobin G
IgM: Immunoglobin M
LPS: Lipopolysaccharide
PCR: Polymerase chain reaction
RBPT: Rose Bengal plate test
R-LPS: Rough lipopolysaccharide
S-LPS: Smooth lipopolysaccharide
USA: United States of America
WHO: World Health Organization
WOAH: Office of International Epizootics World Organization for Animal Health
